# One billion doses and WHO prequalification of nOPV2: Implications for the global polio situation and beyond

**DOI:** 10.1371/journal.pgph.0002920

**Published:** 2024-02-09

**Authors:** Ananda S. Bandyopadhyay, Laura V. Cooper, Simona Zipursky

**Affiliations:** 1 Bill & Melinda Gates Foundation, Seattle, Washington, United States of America; 2 Department of Infectious Disease Epidemiology, Imperial College London, London, United Kingdom; McGill University, CANADA

With the COVID-19 pandemic abating, the only remaining Public Health Emergency of International Concern is polio [1]. Remarkable progress has been made in reducing the global burden of poliomyelitis since the establishment of the Global Polio Eradication Initiative (GPEI) in 1988. However, achieving and sustaining eradication of all forms of polioviruses is being compromised by two sources of paralytic poliomyelitis: persisting pockets of type 1 wild poliovirus transmission in Afghanistan and Pakistan which reported 6 cases each in 2023 [2] and ongoing outbreaks of circulating vaccine-derived polioviruses (cVDPV) [3]. The latter, particularly those arising from type 2 Sabin oral polio vaccine (OPV) strains, have outnumbered cases of wild-type polio over the last few years, mostly due to outbreaks in the WHO African Region which affected 28 different countries in 2023 [4].

Sabin OPVs, in monovalent (mOPV) or trivalent (tOPV) forms, induce robust humoral and intestinal immunity, but while passing through the vaccinee’s intestine can lose their attenuations and in rare cases can revert to neurovirulence. In settings of persistently poor immunity and low levels of sanitation and hygiene, such reverted OPV strains can establish person-to-person circulation and cause paralytic outbreaks [5]. This risk is more prominent with type 2 poliovirus given there is limited mucosal immunity against this serotype of polio in young children following the 2016 global switch from tOPV (types 1, 2 and 3) to bivalent (types 1 and 3) OPV in essential immunization schedules [6,7].

To mitigate the risk of cVDPV and vaccine-associated paralytic poliomyelitis (VAPP) and retain the advantages of OPV—ease of administration, affordability and favorable impact on interrupting virus transmission—a scientific consortium was established in 2011 to develop a novel oral polio vaccine type 2 (nOPV2). Designed at the molecular level to be more genetically stable than Sabin monovalent OPV type 2 (mOPV2) and to have a lower risk of reverting to neurovirulent variants [8], nOPV2 promises to break the vicious cycle of creating new cVDPV emergences while responding to cVDPV2 outbreaks [9].

**Fig 1:**
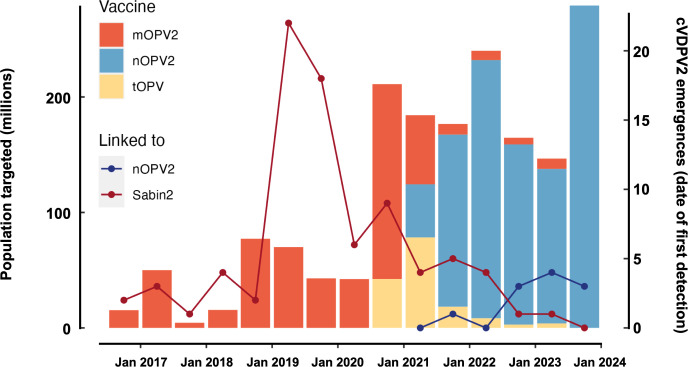
Use of type 2-containing OPV in campaigns linked to emergence of cVDPV linked to Sabin OPV (red line) and nOPV2 (blue line) by six-month periods since the global cessation of the routine use of type 2 OPV, from July 1, 2016 to December 31, 2023 (WHO data on file).

As illustrated in **Fig 1**, increasing numbers of cVDPV2 outbreaks derived from Sabin type 2 vaccines were detected following the tOPV to bOPV switch, peaking in 2019–20 before a gradual decline. To interrupt these outbreaks, there was widespread use of mOPV2 and to a lesser extent tOPV in mass vaccination campaigns between 2016 to 2021. In certain settings this led to seeding of new cVDPV2 emergences derived from Sabin OPV type 2 due to declining population immunity against type 2 poliovirus and insufficient coverage and delays in outbreak response. Given the risk of global spread of cVDPV2 outbreaks, and based on clinical data on the safety, immunogenicity, and genetic stability of nOPV2 generated in a series of clinical trials, the WHO issued an Emergency Use Listing (EUL) recommendation for nOPV2 on November 13, 2021 [10]. The new vaccine, priced the same as the mOPV2 it replaced at less than 20 cents per dose has been rapidly distributed and the downward trend in new cVDPV2 emergences continued following nOPV2 rollout. Approximately 1 billion doses of nOPV2 have been administered between March 13, 2021 and January 1, 2024, compared with approximately 580 and 150 million doses of mOPV2 and tOPV, respectively, from July 1, 2016 to January 1, 2024 (*WHO data on file*). Although some cVDPV2 outbreaks have been linked to nOPV2 itself, the overall trend of new emergences has remained stable since 2021, indicating that the enhanced genetic stability of nOPV2 is limiting the seeding of new cVDPV2 outbreaks despite wide-scale field use [11].

The immediate acceptance of nOPV2 speaks to the recognition of its benefits by national authorities—35 countries have already used the new vaccine, 17 more have approved its use in case of need, and a further 20 have started discussions and preparations should they require it. In the WHO African Region, over 130 supplementary immunization activities (SIAs) using nOPV2 have occurred across 30 countries. However, nOPV2 vaccination campaigns face the same challenges to achieve high coverage and timely implementation in real-world settings as the previous Sabin OPV2 campaigns. As both vaccines are impacted by the heterogeneity in immune response in different sub-populations and given the variability in vaccine coverage across countries multiple campaigns are sometimes necessary with either vaccine. Extensive nOPV2 use in countries such as Nigeria where more than 500 million doses have been administered in multiple campaigns, or the 68 million doses administered in the Democratic Republic of the Congo highlight the importance of improving the quality and reach of vaccination campaigns in high-risk settings.

Although nOPV2 has already had a significant public health impact in reducing the paralytic burden of vaccine-related polio [12], the fundamental challenges of ensuring rapid outbreak response and adequate vaccination coverage through timely, high quality immunization campaigns remain central to the likelihood of eradicating all forms of polio, irrespective of vaccine used. Vaccination strategies need to be aligned with WHO SAGE guidance to further minimize the risk of new cVDPV emergence [13], and moving forward we must ensure a consistent, adequate supply of nOPV2 which will be essential for successful eradication. As nOPV2 is currently only being produced by one manufacturer, ensuring expansion of the manufacturing base to provide sufficient supplies will likely be the single-most important factor in the next phase of nOPV2 use. In addition, considerations for preventative use of nOPV2 may also have to be evaluated in select geographies if outbreaks and risk of transnational spread persist. The process of developing nOPV1 and nOPV3 [14] or multivalent nOPV formulations should be accelerated by using lessons-learned from the development, regulatory approval and rollout of nOPV2 to mitigate cVDPV1 and cVDPV3 risks. Furthermore, while nOPVs are a step forward for outbreak response tools with lower risk of vaccine-related polio, the ultimate strategic goal is to replace all live attenuated OPVs with inactivated polio vaccine (IPV) for essential immunization to completely eliminate the risk of new cVDPV and VAPP. Strengthening essential immunization coverage and targeted use of IPV for outbreak response in specific circumstances, aligned with SAGE guidance, should enhance the probability of interrupting virus transmission and reduce the paralytic burden. Successful outbreak response with stockpiled nOPVs should take the world closer to cessation of all OPV use and allow transition to exclusive use of IPV for essential immunization [15].

Several firsts that have global health implications are attributable to nOPV2: the first vaccine to have a WHO EUL (before the better-known COVID-19 vaccines), on December 27, 2023 it became the first vaccine to transition from a WHO EUL to WHO prequalification. In addition, it represents the only example of a global public-private partnership driving vaccine development, production, supply, rollout, and monitoring, led by the GPEI. Vaccine science advances such as nOPV2 coupled with time-tested principles of vaccination efforts in the field should strengthen the likelihood of global eradication of all forms of polioviruses, an eagerly awaited achievement in global public health.
